# Laparoscopic Versus Open Liver Resection for Colorectal Liver Metastases: A Comprehensive Systematic Review and Meta-analysis

**DOI:** 10.1038/s41598-017-00978-z

**Published:** 2017-04-21

**Authors:** Si-Ming Xie, Jun-Jie Xiong, Xue-Ting Liu, Hong-Yu Chen, Daniel Iglesia-García, Kiran Altaf, Shameena Bharucha, Wei Huang, Quentin M. Nunes, Peter Szatmary, Xu-Bao Liu

**Affiliations:** 1 Department of Gastrointestinal Surgery, West China Hospital of Sichuan University, Cheng du, China; 2 People’s Hospital of Deyang, Deyang, China; 3 Departments of Pancreatic Surgery, West China Hospital of Sichuan University, Chengdu, China; 4 Department of gastrointestinal Surgery, West China Hospital of Sichuan University, Chengdu, China; 5 Clinical Directorate of General Surgery, Royal Liverpool and Broadgreen University Hospitals NHS Trust, Liverpool, UK

## Abstract

The effects of laparoscopic liver resection (LLR) and open liver resection (OLR) on oncological outcomes for colorectal cancer liver metastases (CCLM) remain inconclusive. Major databases were searched from January 1992 to October 2016. Effects of LLR vs OLR were determined. The primary endpoints were oncological outcomes. In total, 32 eligible non-randomized studies with 4697 patients (LLR: 1809, OLR: 2888) were analyzed. There were higher rates of clear surgical margins (OR: 1.64, 95%CI: 1.32 to 2.05, *p* < 0.00001) in the LLR group, without significant differences in disease recurrence, 3- or 5-year overall survival(OS) and disease free survival(DFS) between the two approaches. LLR was associated with less intraoperative blood loss (WMD: −147.46 [−195.78 to −99.15] mL, *P* < 0.00001) and fewer blood transfusions (OR: 0.41 [0.30–0.58], *P* < 0.00001), but with longer operation time (WMD:14.44 [1.01 to 27.88] min*, P* < 0.00001) compared to OLR. Less overall morbidity (OR: 0.64 [0.55 to 0.75], *p* < 0.00001) and shorter postoperative hospital stay (WMD: −2.36 [−3.06 to −1.66] d, *p* < 0.00001) were observed for patients undergoing LLR, while there was no statistical difference in mortality. LLR appears to be a safe and feasible alternative to OLR in the treatment of CCLM in selected patients.

## Introduction

Colorectal cancer (CRC) is one of the commonest cancers, associated with >1.2 million new cases and 600,000 deaths per year globally^1^. In the United States, CRC is the most frequent of gastrointestinal (including liver and pancreatic) cancers and leading cause of death in that group^2^. The liver is the primary site of CRC metastasis, with approximately 14.5% of all patients having developed liver metastases by 5 years following resection of primary tumor with curative intent^3^.

Liver metastases are also often present at first diagnosis of CRC, however they are no longer a contra-indication for surgery. Recent advances in surgical techniques and experience, together with chemotherapeutic regimens have improved median survival for selected patients with colorectal cancer liver metastases (CCLM) to between 35 and 58% at 5-year following liver resection^4–7^, which remains the only prospect of long-term survival^4,8–10^. The established operative approach to CRC metastases is open liver resection (OLR). However, since the introduction of laparoscopic liver resection(LLR) by Gagnerand colleagues in 1992^11^, minimally invasive techniques have found increasing use in the operative management of benign and malignant liver lesions, including CCLM^12^. Several large randomized trials^13–15^ have demonstrated oncological equivalence of laparoscopic versus open resection for the primary tumor in CRC, with reduced postoperative length of hospital stay (PLOS). However, doubts remain about LLR for CCLM due to its technical complexity, the risk of uncontrollable bleeding and gas embolism^16^. Questions also remain in relation to adequacy of surgical margins, port-site metastases, and peritoneal spread^17,18^.

Advances in laparoscopic equipment, techniques, and increasing surgeon experience have meant that outcomes after LLR for benign and malignant primary liver lesions are now equivalent or better than those for OLR^19–21^. As a result, the number of LLRs performed worldwide has increased exponentially in recent years. With regard to CCLM, several retrospective studies^22–26^ have been published demonstrating similar oncological outcomes between LLR and OLR, but LLR is consistently associated with less blood loss, reduced postoperative complications, shortened PLOS, fewer cases of disease recurrence and lower mortality. These studies were all conducted in specialist centers, however, and questions remain regarding selection bias^27^. Nevertheless an international panel of expert surgeons recently stated that LLR was appropriate in the treatment of CCLM^28,29^.

To date, no randomized controlled trials have compared oncological outcomes of LLR and OLR. There have been several meta-analyses^30–34^ consistently showing that LLR improved intra- and post-operative outcomes, but which were inconclusive on oncological outcomes. To address this issue, we conducted the most comprehensive systematic review and meta-analysis using the GRADE system to assess the quality of individual studies and produce the most rigorous analysis of LLR versus OLR in CCLM thus far.

## Results

### Description of included trials in the meta-analysis

**Figure 1:**
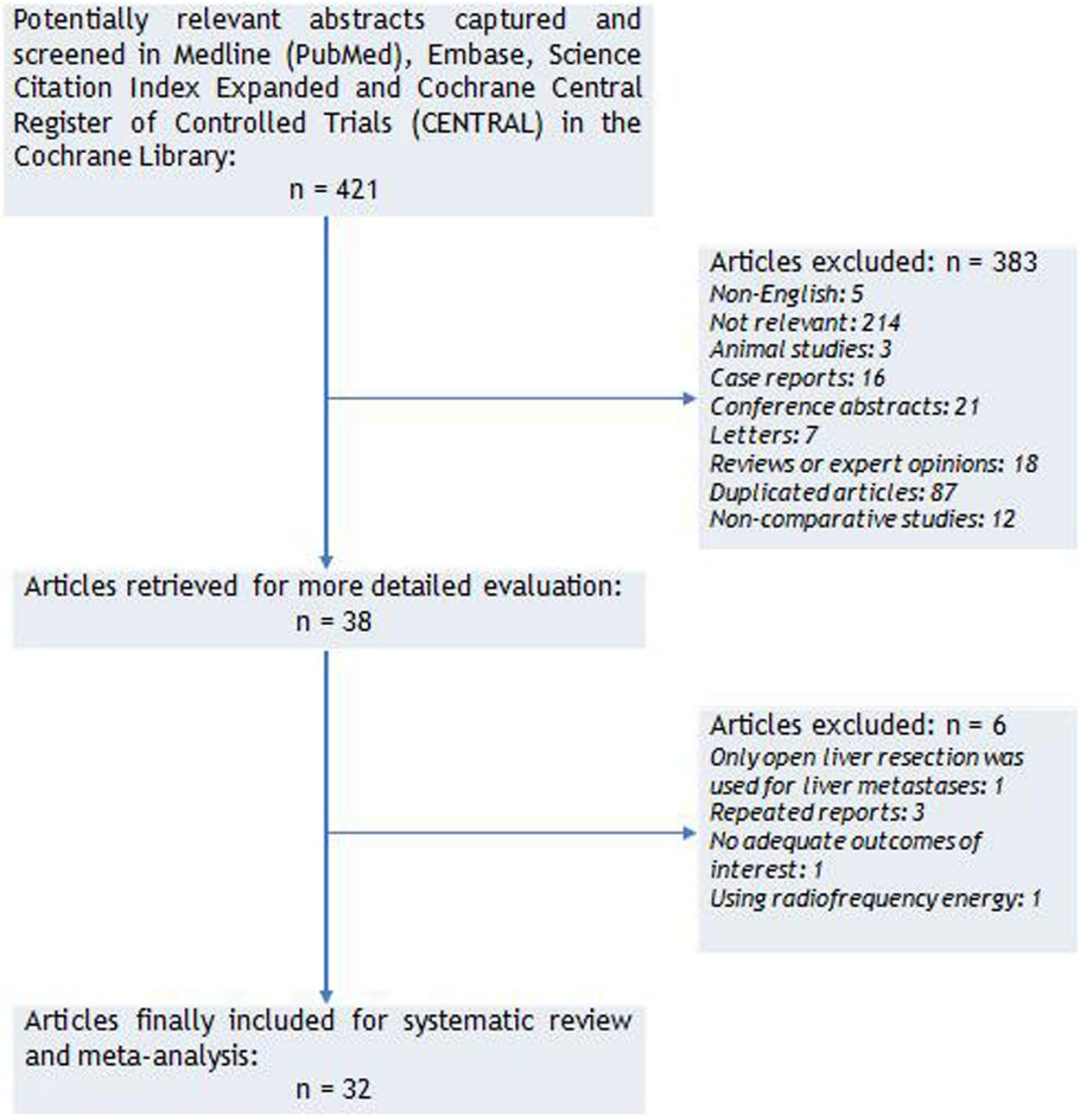
Study selection flow chart according to PRISMA statement.

The search strategy initially generated 421 clinical trials; no randomized clinical trials were identified. Figure 1 details the process of selecting comparative studies using the PRISMA statement for meta-analyses. Initial screening of abstracts led to the exclusion of 383 articles. Besides, six were further excluded by a close scrutiny of remaining articles: 1^35^ only included OLR, 3 duplicates^24,36,37^, 1^38^ did not report outcomes of interest and 1^39^ used radiofrequency ablation. Finally, 32 non-randomized comparative studies were included in our meta-analysis^20,26,27,40–68^.

### Study and patient characteristics

The characteristics of included studies are shown in Table 1. A total of 4697 patients were included: 1809 patients in the LLR and 2888 patients in the OLR group, respectively. The quality assessments of the included studies are given in Supplementary Table S1. The level of GRADE was made according to the GRADE working group recommendations^69^, are summarized in Supplementary Table S2. The sample size of the included studies varied from 14 to 1152 patients. The rate of open conversion ranged from 0% to 15.8%.

**Table 1:** Characteristics of the included studies.

Authors	Year	Country	Design^a^	Group	No. of patients	Age^b^	Sex (M/F)	Tumor size (cm)	No. of tumor resected	Type of LLR^c^	Follow-up (months)	Conversion n (%)	Quality score^d^
Mala *et al*.^27^	2002	Norway	No	LLR	13	68 (55–73)	4/9	2.6 (1–6)	2 (1–7)	Standard	NA	0	5
				OLR	14	59 (24–74)	4/10	3 (1.5–9)	1 (1–4)		NA		
Castaing *et al*.^40^^e^	2009	France	Matched	LLR	60	62 ± 11	37/23	2.2 ± 2.3	2.2 ± 2.3	Standard	32.7 ± 24	6 (10)	6
				OLR	60	62 ± 11	37/23	2.2 ± 2.0	2.2 ± 1.98		33.3 ± 24		
Welsh *et al*.^41^^e^	2010	United Kingdom	No	LLR	266	61.9 (10.4)	161/105	3.3 (1.2)	1 (1–10)	Standard	NA	NA	4
				OLR	886	62.3 (10.1)	324/562	5.3 (3.6)	2 (1–20)		NA		
Chen *et al*.^42^	2011	China	No	LLR	23	55 ± 10	18/5	2.5 ± 0.9	NA	Standard	45.3 (36–72)	0	4
				OLR	18	53 ± 9	14/4	2.3 ± 1.0	NA				
Huh *et al*.^43^^e^	2011	Korea	Matched	LLR	20	63 (36–71)	13/7	2.0 (0.9–5.5)	2 (1–7)	Standard	27.4 (9–73)	0	5
				OLR	20	62 (44–85)	15/5	2.4 (1.0–10.0)	2 (1–8)				
Nguyen *et al*.^20^	2011	United States	No	LLR	24	66.1	10/14	3.0	NA	Standard	26.5	0	5
				OLR	25	65.4	12/13	2.6	NA		29.0		
Cannon *et al*.^44^	2012	United States	Propensity﻿ score﻿	LLR	35	62 (10)	NA	4 (3)	1 ± 1	Standard	NA	0	6
				OLR	140	62 (11)	NA	4 (2)	1 ± 1		NA		
Hu *et al*.^45^	2012	China	Matched	LLR	13	54 ± 10	10/3	3.2 ± 1.0	NA	Standard	16–81	0	5
				OLR	13	53 ± 11	9/4	3.5 ± 0.9	NA				
Topal *et al*.^46^^e^	2012	Belgium	Matched	LLR	20	57.6	10/10	4 (0.4–7)	2 (1–6)	Standard	43.4 (5.5–102)	0	6
				OLR	20	66.0	8/12	3.2 (1–12.5)	2 (1–14)				
Cheung *et al*.^47^^e^	2013	China	Matched	LLR	20	57.5 (42–74)	13:7	1.6 (0.5–4.5)	1 (1–2)	Standard	NA	2 (10)	5
				OLR	40	64 (29–83)	29:11	2.2 (0.5–7)	1 (1–2)		NA		
Doughtie *et al*.^48^^e^	2013	United States	No	LLR	8	59.5	NA	6.8	1.0	Standard	32	0	5
				OLR	76	60.0	NA	7.5	1.5				
Guerron *et al*.^26^^e^	2013	United States	Matched	LLR	40	66.2 ± 1.9	19/21	1.3 ± 0.1	1.3 ± 0.1	Partial	16 (1–51)	0	5
				OLR	40	62.2 ± 1.8	15/25	1.7 ± 0.1	1.7 ± 0.1	HLR			
Inoue *et al*.^49^	2013	Japan	No	LLR	23	66.1 ± 9.6	11/12	2.5 ± 1.1	NA	Standard	NA	0	5
				OLR	24	68.0 ± 9.5	13/11	2.7 ± 0.9	NA		NA		
Iwahashi *et al*.^50^^e^	2014	Japan	Matched	LLR	21	67.5 ± 11.1	16/5	2.4 ± 0.8	1.8 ± 1.1	Partial	NA	0	6
				OLR	21	68.2 ± 10.4	14/7	2.6 ± 0.8	2.1 ± 1.2	HLR	NA		
Jung *et al*.^51^^e^	2014	Korea	Matched	LLR	24	60.0 (43–75)	13/11	2.5 (0.3–7.0)	15/9^h^	Standard	NA	0	4
				OLR	24	60.0 (37–80)	17/7	2.5 (0.9–9.5)	11/13^h^		NA		
Kubota *et al*.^52^	2014	Japan	No	LLR	43	64.4 ± 11.4	22/21	NA	27/15/1^i^	Partial	NA	0	4
				OLR	62	65.5 ± 11.5	40/22	NA	23/27/12^i^	HLR	NA		
Montalti *et al*.^53^	2014	Italy	Matched	LLR	57	61.7 ± 11	20/37	NA	NA	Standard	40.9 (10–1.2)	9 (15.8)	6
				OLR	57	63.5 ± 10	23/34	NA	NA		53.7 (2.6–3.2)		
Qiu *et al*.^54^	2014	China	Matched	LLR	24	45.9 ± 9.8	10/14 12/13	2.7 ± 2.1	14/10^j^	Standard	30.6 (6–37)	2 (8.3)	6
				OLR	25	45.5 ± 9.3		2.9 ± 1.5	9/16^j^		32.4 (8–40)		
Takasu *et al*.^55^	2014	Japan	Matched	LLR	7	74 ± 12	3/4	1.4 ± 0.8	NA	Standard	31.5 ± 33.5	0	5
				OLR	7	62 ± 5	3/4	1.5 ± 1.1	NA		41.2 ± 27.2		
Allard *et al*.^56^^e^	2015	France	Propensity score	LLR	153	NA	61/90	NA	1458/775^h^	Partial	NA	NA	4
				OLR	153	NA	62/91	NA	149/27^h^	HLR and RLR	NA		
Beppu *et al*.^57^	2015	Japan	Propensity score	LLR	171	NA	107/64	4/167 f	127/43/1^i^	Partial	NA	NA	6
				OLR	342	NA	215/126	8/334/f	251/89/2^i^	HLR and hybrid	NA		
de’Angelis *et al*.^58^^e^	2015	France	Propensity score	LLR	52	63 (32–81)	25/27 23/29	2.6 (1.5–6)	1 (1–4)	Standard	58.6–44.4	3 (5.8)	6
				OLR	52	63 (46–83)		3 (1.5–5.2)	2 (1–12)		54.1–43.4		
Hasegawa *et al*.^59^	2015	Japan	No	LLR	100	67 (24–91)	64/36	2.3 (7–9.5)	1 (1–8)	Partial	NA	1 (1)	4
				OLR	68	65 (37–83)	43/25	3.5 (1.1–16)	2 (1–12)	HLR andhybrid	NA		
Langella *et al*.^60^	2015	Italy	Matched	LLR	37	63 (37–86)	25/12	NA	1 (1–4)	Standard	35.7	NA	5
				OLR	37	65 (50–81)	25/12	NA	1 (1–4)		47.9		
Lin *et al*.^61^^e^	2015	China	Propensity score	LLR	36	57.5 ± 7.3	19/17	3.7 ± 2.0	1.9 ± 1.2	Partial	43.4 (11–69)	NA	6
				OLR	36	57.4 ± 10.4	21/15	4.2 ± 2.2	2.1 ± 1.0	RLR			
Nachmany *et al*.^62^	2015	Israel	No	LLR	42	64.5 ± 12	22/20	3.3 ± 2.2	1.75 ± 1.16	Standard	NA	5 (11.9)	4
				OLR	132	62 ± 11.9	70/62	3.5 ± 2.8	2.82 ± 2.81		NA		
Tohme *et al*.^63^	2015	United States	Matched	LLR	66	62.1 (11.2)	37/29	2.2 (1.5–3.0)	1 (1–2)	Partial	NA	3 (4)	5
				OLR	66	62.5 (12.3)	43/23	2.6 (2.0–3.5)	2 (1–3)	HLR and RLR	NA		
Lewin *et al*.^64^^e^	2016	Australia	Propensity score	LLR	146	63.05	NA	NA	NA	Partial	36	NA	5
				OLR	140	61.35	NA	NA	NA	HLR andhybrid			
Ratti *et al*.^65^^e^	2016	Italy	Propensity score	LLR	25	60 (37–80)	14/11	2.9 (0.5–11)	2 (1–6)	LLR	37 (15–75)	1 (4)	5
				OLR	50	62 (35–81)	27/23	3.4 (0.9–12)	2 (1–7)				
Tranchart *et al*.^66^^e^	2016	Japan	Propensity score	LLR	89	66.6 ± 10.8	47/42	2.9 ± 1.9	1.4 ± 0.6	Partial	26 (1–94)	6 (7)	5
				OLR	89	65.0 ± 9.4	49/40	2.8 ± 2.0	1.5 ± 0.7	HLR	26 (1–100)		
Untereiner *et al*.^67^^e^	2016	France	Propensity score	LLR	18	68.0 (50.8–74.8)	5/13	2.8 (2.0–4.6)	1 (1–2)	Partial	5.4 (1.4–11.6)	1 (5.6)	6
				OLR	18	63.5 (59.0–67.5)	7/11	4.3 (2.3–11.5)	1 (1–2)	RLR			
Cipriani *et al*.^68^^e^	2016	United Kingdom	Propensity score	LLR	133	85/48^g^	79/54	21/112f	54/79^h^	Standard	23	13 (9.8)	4
				OLR	133	80/53^g^	83/50	106/27f	56/77^h^		30		

*LLR*: laparoscopic liver resection, *NA*: not available, *HLR*: hand-assisted liver resection, *RLR*: robotic-assisted liver resection.

^a^All studies were retrospective observational study.

^b^Age is expressed median (range), mean ± standard deviation, median, or mean.

^c^Some studies included a portion of LLR managed with HLR, RLR, or hybrid.

^d^Assessed by modified Newcastle-Ottawa Scale.

^e^Data were analyzed from prospective databases in these studies.

^f^No. of patients with tumor size < 5 and ≥ 5 cm.

^g^No. of patients with age ≤ 70 and >70 years.

^h^No. of liver lesions: single or multiple.

^i^No. of liver lesions:1 or 2–4 or ≥ 5.

^j^No. of liver lesions:1 or ≥ 2.

### Meta-analysis results

**Figure 2:**
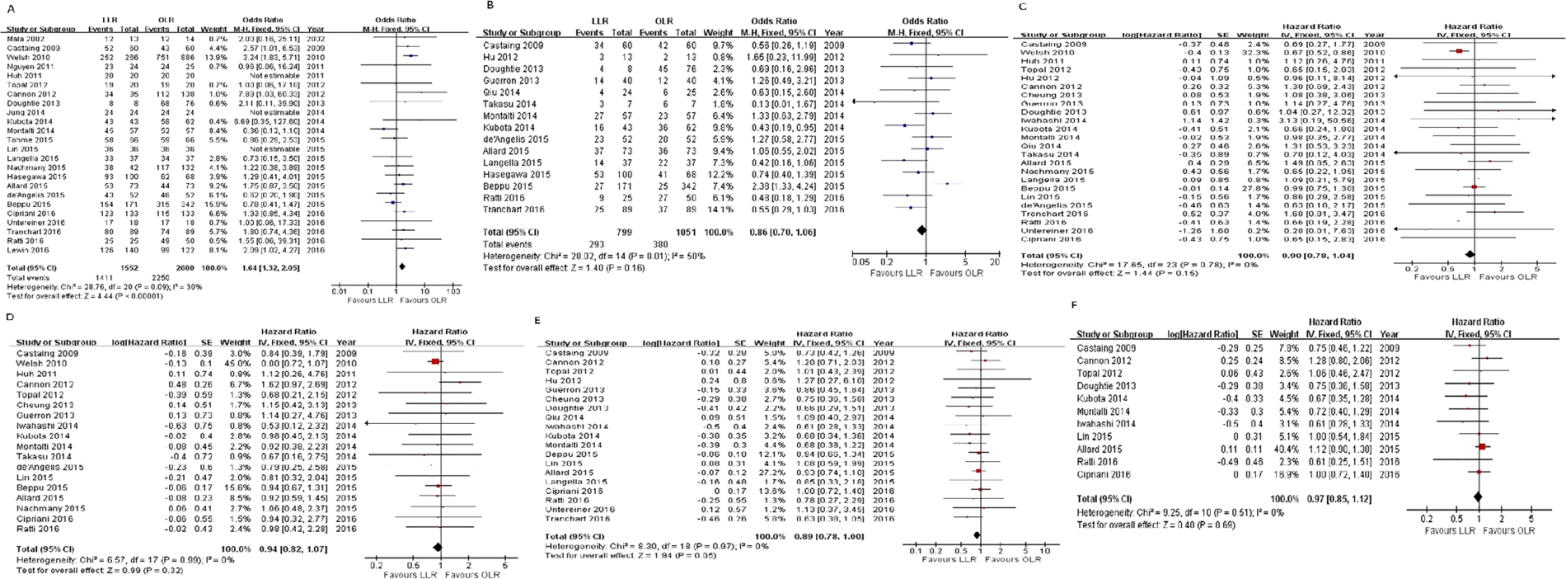
(**A**) Forest plot of negative surgical margin between two groups. (**B**) Forest plot of recurrence between two groups. (**C**). Forest plot of 3-year OS between two groups. (**D**) Forest plot of 5-year OS between two groups. (**E**) Forest plot of 3-year DFS between two groups. (**F**) Forest plot of 5-year DFS between two groups.

Results of individual analyses are shown in Fig. 2 and Supplementary Fig. S1.

Primary outcomes: LLR had higher rates of clear resection margins (R0) compared to OLR in the 24 studies reporting this outcome (*n* = 4152; OR: 1.64 [1.32 to 2.05], *p* < 0.00001; *I*^*2*^ = 30%; GRADE: moderate). However, we did not find any statistically significant differences in tumor recurrence (*n* = 1850; OR: 0.86 [0.70 to 1.06], *p* = 0.16; *I*^*2*^ = 50%;moderate), 3-year OS (HR: 0.90 [0.78 to 1.04], *p* = 0.15; moderate) and 5-year OS (HR: 0.94 [0.82 to 1.07], *p* = 0.32; moderate), or 3-year DFS (HR: 0.89 [0.78 to 1.00], *p* = 0.05;moderate) and 5-year DFS(HR: 0.97 [0.85 to 1.12], *p* = 0.69; moderate). There was no heterogeneity among studies reporting OS and DFS (all *I*^*2*^ = 0).

Secondary outcomes: 24 studies reported mean operation time, demonstrating longer operative times on average in the LLR group (*n* = 2441; WMD: 14.44 min [1.01 to 27.88]; *p* < 0.00001; *I*^*2*^ = 71%; very low); 26 studies estimated overall less blood was lost intra-operatively in LLR compared to OLR (*n* = 2700; WMD: −147.46 mL [−195.78 to −99.15]; *p* < 0.00001; *I*^*2*^ = 91%; very low); furthermore, the rate of blood transfusion was lower in the LLR group in 15 reporting studies (*n* = 1807; OR: 0.41 [0.30 to 0.58], *p* < 0.00001; *I*^*2*^ = 0%; moderate); PLOS, reported in 26 studies, was shorter in LLR patients(*n* = 3735; WMD: −2.36d [−3.06 to −1.66]; *p* < 0.00001;*I*^*2*^ = 78%; very low); similarly, patients undergoing LLR experienced less overall morbidity (30 trials; *n* = 4197; OR: 0.64 [0.55 to 0.75]; *p* < 0.00001; moderate), but no statistically significant difference in mortality (28 trials; *n* = 4277; OR: 0.98 [0.58 to 1.70], *p* = 0.98; moderate).There was no heterogeneity among studies reporting overall morbidity and mortality (both *I*^*2*^ = 0).

### Sensitivity and subgroup analysis

Results from sensitivity and subgroup analyses are summarized in Table 2.

**Table 2:** The result of subgroup and sensitivity analysis.

Outcomes of interest	No. of studies	No. of patients	WMD/OR/HR (95% CI)	*p-value*	Heterogeneity *p*-value	*I*^*2*^ (%)
Studies with high quality						
Negative surgical margin	17	2093	1.26 (0.94,1.69)	0.12	0.22	21
Recurrence	12	1431	0.85 (0.56,1.27)	0.43	0.01	54
3-OS	19	1896	1.03 (0.85,1.25)	0.76	0.99	0
5-OS	13	1449	1.00 (0.81,1.23)	0.98	0.93	0
3-DFS	16	1738	0.85 (0.72,1.00)	0.05	0.96	0
5-DFS	8	722	0.87 (0.70,1.08)	0.21	0.61	0
Studies with propensity score matching						
Negative surgical margin	10	1825	1.51 (1.12,2.05)	0.007	0.22	25
Recurrence	5	1016	1.00 (0.55,1.82)	0.99	0.006	72
3-OS	9	1725	1.08 (0.88,1.32)	0.48	0.65	0
5-OS	7	1511	1.02 (0.82,1.26)	0.86	0.68	0
3-DFS	8	1621	0.94 (0.81,1.09)	0.43	0.81	0
5-DFS	5	894	1.08 (0.92,1.26)	0.36	0.66	0
Studies with case matching						
Negative surgical margin	8	673	1.11 (0.67,1.83)	0.69	0.11	44
Recurrence	7	477	0.78 (0.53,1.13)	0.19	0.24	25
3-OS	11	659	0.99 (0.67,1.46)	0.97	1.00	0
5-OS	8	496	0.87 (0.59,1.28)	0.48	0.99	0
3-DFS	9	605	0.78 (0.61,1.01)	0.06	0.98	0
5-DFS	4	316	0.75 (0.55,1.03)	0.07	0.81	0
Studies with sample size >50						
Negative surgical margin	19	3961	1.65 (1.32,2.06)	<0.00001	0.04	40
Recurrence	12	1761	0.84 (0.59,1.17)	0.30	0.008	56
3-OS	17	3652	0.89 (0.77,1.03)	0.13	0.50	0
5-OS	14	3316	0.95 (0.83,1.08)	0.41	0.96	0
3-DFS	14	2222	0.88 (0.78,1.00)	0.06	0.91	0
5-DFS	9	1317	0.99 (0.86,1.13)	0.84	0.45	0
Studies in Eastern countries						
Negative surgical margin	7	1124	1.17 (0.74,1.86)	0.49	0.27	23
Recurrence	8	1133	0.83 (0.47,1.44)	0.50	0.005	65
3-OS	10	1099	1.04 (0.83,1.30)	0.73	0.93	0
5-OS	7	846	0.92 (0.71,1.20)	0.56	0.98	0
3-DFS	8	1045	0.83 (0.67,1.03)	0.09	0.79	0
5-DFS	3	219	0.77 (0.52,1.13)	0.18	0.54	0
Studies with simultaneous colorectal and liver resection						
Negative surgical margin	5	413	1.78 (0.76,4.19)	0.19	0.93	0
Recurrence	4	293	0.54 (0.33,0.88)	0.01	0.48	0
3-OS	6	405	1.13 (0.70,1.81)	0.62	0.79	0
5-OS	4	187	0.89 (0.52,1.51)	0.66	0.95	0
3-DFS	4	351	0.81 (0.56,1.15)	0.24	0.55	0
5-DFS	2	147	0.86 (0.52,1.42)	0.55	0.38	0
Studies without HLR, RLR, or hybrid						
Negative surgical margin	15	2540	1.89 (1.39,2.57)	<0.0001	0.06	41
Recurrence	9	660	0.75 (0.54,1.05)	0.09	0.31	15
3-OS	16	2567	0.78 (0.64,0.94)	0.01	0.95	0
5-OS	12	2334	0.94 (0.80,1.10)	0.46	0.89	0
3-DFS	11	1083	0.90 (0.74,1.09)	0.28	0.94	0
5-DFS	7	874	0.92 (0.75,1.12)	0.42	0.58	0

*LLR* laparoscopic liver resection, *OLR* open liver resection, *WMD* weight mean differences, *OR* odds ratios; *HR* hazard ratios; *CI* confidence intervals, *OS* overall survival, *DFS* disease-free survival, *HLR* hand-assisted liver resection, *RLR* robotic-assisted liver resection.

All subgroup and sensitivity analyses did not reveal significant changes of 5-year OS or 3- and 5-year DFS, apart from that tumor recurrence was reduced in studies with sample size >50 or performed in Eastern countries and 3-OS was improved in studies using LLR assisted with other modalities. Analyses for studies with propensity score matching, cases >50 and LLR assisted with other modalities did not change primary meta-analysis results or statistical heterogeneity for negative surgical margin. However, this outcome measure was not significant anymore when in subgroup analyses of studies with higher quality or studies with simultaneous colorectal and liver resection or studies in Eastern countries.

### Meta-regression analysis

None of the included covariates had any significant impact on heterogeneity (Supplementary Table S3).

### Publication bias

**Figure 3:**
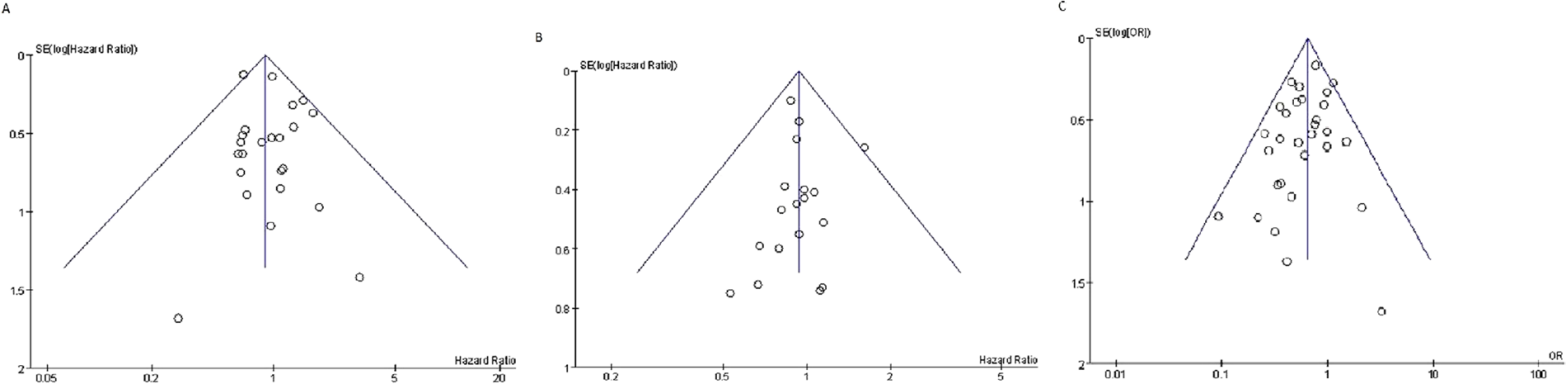
Funnel plots for publication bias. The funnel plot revealed no publication bias. (**A**) 3-OS; (**B**) 5-OS; (**C**) overall morbidity.

The funnel plots were based on the 3-, 5-year OS and overall morbidity, which is shown in Fig. 3. As no study lies outside the limits of the 95%CI, there was no evidence of publication bias.

## Discussion

This study provides the most robust evidence to date that LLR is a viable alternative to OLR in the treatment of CCLM in select patients. Using the GRADE system of qualifying evidence, we can report with relative confidence that post-operative mortality was equivalent in both groups, but overall morbidity was almost halved by LLR. Importantly, there were also no statistically significant differences in recurrence rates, 3- and 5- year OS or DFS. If at all, there was a trend towards a benefit from LLR in our analysis. This is entirely supported by our finding that there were a greater proportion of clear resection margins (R0) in the LLR group. While it is theoretically possible that operative factors such as magnification or extra caution when using LLR techniques indeed led to an improved resection rate, it is more likely that this represents selection bias. Therefore, it is probable that larger, more aggressive tumors were either not even attempted or converted to OLRs and, thus, artificially enhancing the pool of OLRs with high-risk malignancies.

Indeed, there is currently no consensus in the literature as to which patients with CCLM are suitable for LLR, although LLR has been performed for all liver segments in this context. Small tumors in the left lateral segments of the liver^70,71^ and patients with limited tumor burden (2 metastases or fewer)^33^ have been reported as advantageous in LLR. Similarly, patients with tumors involving the inferior vena cava, left or right portal veins, roots of any of the hepatic veins, or patients with multifocal or bilobar tumors are not good candidates for a minimally invasive liver resection. As all studies included in our analysis were non-randomised comparison studies, and the LLR groups had fewer numbers of tumor resected, this is further indicative of selection bias.

Our analysis agrees with previous meta-analyses that LLR is associated with longer surgery (by around 15 minutes), less blood loss (about 150 mL) and shorter PLOS (around 2 days). Interestingly, GRADE analysis rates the quality of this conclusion as ‘very low’, based on very high heterogeneity between studies, higher than expected variances in the sample populations and unmeasured confounding factors. One of the likely sources of significant heterogeneity between studies is the constantly and rapidly evolving nature of LLR. Modifications of equipment and technique, such as the use of intraoperative ultrasonography, ultrasonic dissection, microwave coagulators, endoscopic linear staplers, laparoscopic CUSA and vascular staplers, have simplified LLR and addressed concerns about major intra-operative hemorrhage^72–75^. Due to these methological difficulties as well as the modest improvements in the outcome parameters it would be unreasonable to offer one type of surgery over the other on this basis alone.

Based on our GRADE analysis, one secondary outcome stands out as being both statistically significant, consistent amongst studies and potentially clinically significant. People in the LLR were less than half as likely as those in the OLR group to receive a blood transfusion in the perioperative period. This could be a combination of a lower intra-operative blood loss as well as reduced risk of abdominal wall/muscular bleeding in the post-operative period. This is potentially of clinical significance due to the ongoing debate whether blood transfusions (through immunogenicity or otherwise) contribute to worse long-term survival in colorectal cancer^76–79^.

Our subgroup and sensitivity analysis supports our conclusions. Propensity score matching allowed us to take known confounders into account^80^, considered to approach the accuracy of a randomized controlled trial, however reliant on knowing the confounders^81^. Nevertheless, the importance of all disease- and patient- relevant confounders on the choice of surgery in CCLM can only be determined in a well-designed randomized controlled trial, insisting on homogeneity of tumor characteristics, operative technique and use of adjuvant therapy.

Based on current evidence, LLR is at least as safe as OLR in the treatment of CCLM in specialist centers and has the potential to significantly reduce morbidity in this population.

## Methods

### Literature search and study selection

PubMed (Medline), EMBASE and Science Citation Index Expanded and Cochrane Central Register of Controlled Trials (CENTRAL) in the Cochrane Library were searched systematically for all articles published as full papers in the English language from January 1992 to October 2016 comparing LLR and OLR for CCLM. The following medical search headings (MeSH) and keywords were used: “laparoscopy” or “laparoscopic” or “robotic” or “robot-assisted” or “minimally invasive surgery” and “hepatectomy” or “liver resection” or “hepatic resection” and “colorectal cancer” or “colorectal neoplasm” or “colorectal liver metastases”. Reference lists of selected articles were also examined to find relevant studies which were not identified during the initial database searches. The database searching was supplemented with manual searching for reference lists of obtained articles, unpublished studies, and conference abstracts. We contacted the authors for full-text or original data of their investigations where required. Final inclusion of articles was determined by consensus from two authors (S.M.X. and J.J.X.); when this failed, a third author adjudicated (X.T.L.).The whole process of this study followed the Preferred Reporting Items for Systematic Reviews and Meta-Analyses (PRISMA) statement^82^.

### Inclusion and exclusion criteria

Three authors (S.M.X., J.J.X. and X.T.L.) identified and screened the aforementioned databases for potentially eligible studies.

#### Inclusion criteria

(1) adult patients with CCLM; (2) clear documentation of the operative techniques as OLR or LLR with either hand-assisted, robot-assisted, or hybrid; (3) studies with at least one of the outcomes of interest mentioned; (4) where multiple studies came from the same institute and/or authors, either the one of higher quality or the most recent publication was included in the analysis.

#### Exclusion criteria

(1) abstracts, letters, editorials, expert opinions, case reports, reviews and studies lacking control groups; (2) studies with no clearly reported outcomes of interest; (3) studies including patients with other types of malignant liver tumors, (4) non-comparative studies, using only OLR or LLR; (5) animal studies (6) study with radiofrequency energy was used.

### Outcomes of interest

Primary outcomes: negative surgical resection margin, recurrence, 3-and 5-year overall survival (OS), 3- and 5-year disease-free survival (DFS). In some cases, 5-year OS or DFS included these patients with a follow-up between 3–5 years. Secondary outcomes: operative time, intraoperative blood loss and need for blood transfusion; overall morbidity, mortality and PLOS.

### Data extraction and quality assessment

Data were extracted by three independent observers (S.M.X., J.J.X. and X.T.L.) using standardized forms. The recorded data included patient and study characteristics and surgical details. For non-randomized controlled studies, a modification of the Newcastle-Ottawa Scale (NOS)^83,84^ was used for selection, comparability and outcome assessment. Studies valued more than four (of six) stars were recognized as being moderate to high quality.

### Statistical analysis

Meta-analysis was performed using Review Manager Version 5.0 software (The Cochrane Collaboration, Oxford, UK). For continuous and categorical variables, treatment effects were expressed as weighted mean differences (WMD) and odds ratios (OR) with corresponding 95% confidence intervals (CI), respectively. For survival analysis, we extracted data from survival curve referring to method reported in previous study, and hazard ratios (HR) were used for quantitative analysis^85^. An HR of < 1 represented a survival benefit favoring the LLR group and *p* values < 0.05 indicated statistical significance. Medians were converted to means using a previously described methodology^86^. Heterogeneity was assessed by *I*^*2*^ with *p* < 0.1 taken as significant^87^. An *I*^*2*^ value of < 25% was defined to denote low heterogeneity, a value between 25 and 50% was defined as moderate heterogeneity and a value of >50% was considered to be of high heterogeneity. The fixed-or random-effects model was used as appropriate^88^. Subgroup and sensitivity analyses were undertaken by only including studies with high quality, propensity score matching, case matching, sample size >50, conducted in Eastern countries, simultaneous colorectal and liver resection, and LLR assisted with other modalities. Meta-regression analyses assessed impact of publication year, sex, age, study design and tumor size on summary estimates using Stata SE Version 13 Software (StataCorp LP, Texas, USA); *P* < 0.05 was considered significant. Funnel plots were constructed to evaluate potential publication bias based on the 3-, 5-year OS and overall morbidity^89^.

## Electronic supplementary material

SUPPLEMENTARY INFO

## References

[1] Brenner H, Kloor M, Pox CP (2014). Colorectal cancer. Lancet..

[2] Peery AF (2015). Burden of Gastrointestinal, Liver, and Pancreatic Diseases in the United States. Gastroenterology..

[3] Manfredi S (2006). Epidemiology and management of liver metastases from colorectal cancer. Ann Surg..

[4] Choti MA (2002). Trends in long-term survival following liver resection for hepatic colorectal metastases. Ann Surg..

[5] Abdalla EK (2004). Recurrence and outcomes following hepatic resection, radiofrequency ablation, and combined resection/ablation for colorectal liver metastases. Ann Surg..

[6] Leonard GD, Brenner B, Kemeny NE (2005). Neoadjuvant chemotherapy before liver resection for patients with unresectable liver metastases from colorectal carcinoma. J Clin Oncol.

[7] Abdalla EK (2006). Improving resectability of hepatic colorectal metastases: expert consensus statement. Ann Surg Oncol..

[8] Nordlinger B (2008). Perioperative chemotherapy with FOLFOX4 and surgery versus surgery alone for resectable liver metastases from colorectal cancer (EORTC Intergroup trial 40983): a randomised controlled trial. Lancet..

[9] Snoeren N, Voest E, van Hillegersberg R (2008). Surgery vs surgery and chemotherapy for colorectal liver metastases. Lancet..

[10] Fong, Y., Fortner, J., Sun, R. L., Brennan, M. F. &Blumgart, L. H. Clinical score for predicting recurrence after hepatic resection for metastatic colorectal cancer: analysis of 1001 consecutive cases. *Ann Surg*. **230**, 309–318, 10.1097/00000658-199909000-00004, discussion 318–321 (1999). 10.1097/00000658-199909000-00004 PMC1420876 10493478

[11] Gagner M, Rheault M, Dubuc J (1992). Laparoscopic partial hepatectomy for liver tumor. Surgical Endoscopy.

[12] Koffron A, Geller D, Gamblin TC, Abecassis M (2006). Laparoscopic liver surgery: Shifting the management of liver tumors. Hepatology..

[13] Lacy AM (2002). Laparoscopy-assisted colectomy versus open colectomy for treatment of non-metastatic colon cancer: a randomised trial. Lancet..

[14] Clinical Outcomes of Surgical Therapy Study G. A comparison of laparoscopically assisted and open colectomy for colon cancer. *N Engl J Med*. **350**, 2050-2059, doi:10.1056/NEJMoa032651 (2004). 10.1056/NEJMoa032651 15141043

[15] Guillou PJ (2005). Short-term endpoints of conventional versus laparoscopic-assisted surgery in patients with colorectal cancer (MRC CLASICC trial): multicentre, randomised controlled trial. Lancet..

[16] Gagner M, Rogula T, Selzer D (2004). Laparoscopic liver resection: benefits and controversies. Surg Clin North Am.

[17] Martin, R. C., Scoggins, C. R., & McMasters, K. M. Laparoscopic hepatic lobectomy: advantages of a minimally invasive approach. *J Am Coll Surg*. **210**, 627–634, 634–626, doi:10.1016/j.jamcollsurg.2009.12.022 (2010). 10.1016/j.jamcollsurg.2009.12.022 20421019

[18] Reddy SK, Tsung A, Geller DA (2011). Laparoscopic liver resection. World J Surg.

[19] Sarpel U (2009). Outcome for patients treated with laparoscopic versus open resection of hepatocellular carcinoma: case-matched analysis. Ann Surg Oncol..

[20] Nguyen KT (2011). Comparative benefits of laparoscopic vs open hepatic resection: a critical appraisal. Arch Surg..

[21] Nguyen KT, Gamblin TC, Geller DA (2009). World review of laparoscopic liver resection-2,804 patients. Ann Surg..

[22] Pilgrim CH, To H, Usatoff V, Evans PM (2009). Laparoscopic hepatectomy is a safe procedure for cancer patients. HPB (Oxford).

[23] Nguyen KT (2009). Minimally invasive liver resection for metastatic colorectal cancer: a multi-institutional, international report of safety, feasibility, and early outcomes. Ann Surg..

[24] Abu Hilal M, Underwood T, Zuccaro M, Primrose J, Pearce N (2010). Short- and medium-term results of totally laparoscopic resection for colorectal liver metastases. Br J Surg.

[25] Feroci F (2013). Laparoscopic surgery improves postoperative outcomes in high-risk patients with colorectal cancer. Surg Endosc..

[26] Guerron AD (2013). Laparoscopic versus open resection of colorectal liver metastasis. Surg Endosc..

[27] Mala T (2002). A comparative study of the short-term outcome following open and laparoscopic liver resection of colorectal metastases. Surg Endosc..

[28] Buell JF (2009). The international position on laparoscopic liver surgery: The Louisville Statement, 2008. Ann Surg..

[29] Wakabayashi G (2015). Recommendations for laparoscopic liver resection: a report from the second international consensus conference held in Morioka. Ann Surg..

[30] Zhou Y, Xiao Y, Wu L, Li B, Li H (2013). Laparoscopic liver resection as a safe and efficacious alternative to open resection for colorectal liver metastasis: a meta-analysis. BMC Surg..

[31] Luo LX, Yu ZY, Bai YN (2014). Laparoscopic hepatectomy for liver metastases from colorectal cancer: a meta-analysis. J Laparoendosc Adv Surg Tech A..

[32] Wei M (2014). Laparoscopic versus open hepatectomy with or without synchronous colectomy for colorectal liver metastasis: a meta-analysis. PLoS One..

[33] Schiffman SC, Kim KH, Tsung A, Marsh JW, Geller DA (2015). Laparoscopic versus open liver resection for metastatic colorectal cancer: a metaanalysis of 610 patients. Surgery..

[34] Hallet J (2016). Short and long-term outcomes of laparoscopic compared to open liver resection for colorectal liver metastases. Hepatobiliary Surg Nutr..

[35] Ratti F, Catena M, Di Palo S, Staudacher C, Aldrighetti L (2015). Laparoscopic Approach for Primary Colorectal Cancer Improves Outcome of Patients Undergoing Combined Open Hepatic Resection for Liver Metastases. World J Surg..

[36] Qiu J, Chen S, Pankaj P, Wu H (2013). Laparoscopic hepatectomy for hepatic colorectal metastases — a retrospective comparative cohort analysis and literature review. PLoS One..

[37] Topal B (2012). Minimally invasive liver surgery for metastases from colorectal cancer: oncologic outcome and prognostic factors. Surg Endosc..

[38] Fretland AA (2015). Inflammatory Response After Laparoscopic Versus Open Resection of Colorectal Liver Metastases: Data From the Oslo-CoMet Trial. Medicine (Baltimore)..

[39] Vavra P (2015). Colorectal cancer liver metastases: laparoscopic and open radiofrequency-assisted surgery. Wideochir Inne Tech Maloinwazyjne..

[40] Castaing D (2009). Oncologic results of laparoscopic versus open hepatectomy for colorectal liver metastases in two specialized centers. Ann Surg..

[41] Welsh FK, Tekkis PP, John TG, Rees M (2010). Open liver resection for colorectal metastases: better short- and long-term outcomes in patients potentially suitable for laparoscopic liver resection. HPB (Oxford)..

[42] Chen KY, Xiang GA, Wang HN, Xiao FL (2011). Simultaneous laparoscopic excision for rectal carcinoma and synchronous hepatic metastasis. Chin Med J (Engl)..

[43] Huh JW, Koh YS, Kim HR, Cho CK, Kim YJ (2011). Comparison of laparoscopic and open colorectal resections for patients undergoing simultaneous R0 resection for liver metastases. Surg Endosc..

[44] Cannon RM, Scoggins CR, Callender GG, McMasters KM, Martin RC (2012). Laparoscopic versus open resection of hepatic colorectal metastases. Surgery..

[45] Hu MG, Ou-yang CG, Zhao GD, Xu DB, Liu R (2012). Outcomes of open versus laparoscopic procedure for synchronous radical resection of liver metastatic colorectal cancer: a comparative study. Surg Laparosc Endosc Percutan Tech..

[46] Topal H, Tiek J, Aerts R, Topal B (2012). Outcome of laparoscopic major liver resection for colorectal metastases. Surg Endosc..

[47] Cheung TT (2013). Outcome of laparoscopic versus open hepatectomy for colorectal liver metastases. ANZ J Surg..

[48] Doughtie CA (2013). Laparoscopic hepatectomy is a safe and effective approach for resecting large colorectal liver metastases. Am Surg..

[49] Inoue Y (2013). Short-term results of laparoscopic versus open liver resection for liver metastasis from colorectal cancer: a comparative study. Am Surg..

[50] Iwahashi S (2014). Laparoscopic hepatic resection for metastatic liver tumor of colorectal cancer: comparative analysis of short- and long-term results. Surg Endosc..

[51] Jung KU (2014). Outcomes of simultaneous laparoscopic colorectal and hepatic resection for patients with colorectal cancers: a comparative study. J Laparoendosc Adv Surg Tech A..

[52] Kubota Y (2014). Efficacy of laparoscopic liver resection in colorectal liver metastases and the influence of preoperative chemotherapy. World J Surg Oncol..

[53] Montalti R (2014). Laparoscopic liver resection compared to open approach in patients with colorectal liver metastases improves further resectability: Oncological outcomes of a case-control matched-pairs analysis. Eur J Surg Oncol..

[54] Qiu J, Chen S, Pankaj P, Wu H (2014). Laparoscopic hepatectomy is associated with considerably less morbidity and a long-term survival similar to that of the open procedure in patients with hepatic colorectal metastases. Surg Laparosc Endosc Percutan Tech..

[55] Takasu C (2014). Benefits of simultaneous laparoscopic resection of primary colorectal cancer and liver metastases. Asian J Endosc Surg..

[56] Allard MA (2015). Early and Long-term Oncological Outcomes After Laparoscopic Resection for Colorectal Liver Metastases: A Propensity Score-based Analysis. Ann Surg..

[57] Beppu T (2015). Long-term and perioperative outcomes of laparoscopic versus open liver resection for colorectal liver metastases with propensity score matching: a multi-institutional Japanese study. J Hepatobiliary Pancreat Sci..

[58] de’Angelis N (2015). Laparoscopic versus open resection for colorectal liver metastases: a single-center study with propensity score analysis. J Laparoendosc Adv Surg Tech A..

[59] Hasegawa Y (2015). Long-term outcomes of laparoscopic versus open liver resection for liver metastases from colorectal cancer: A comparative analysis of 168 consecutive cases at a single center. Surgery..

[60] Langella S (2015). Oncological safety of ultrasound-guided laparoscopic liver resection for colorectal metastases: a case-control study. Updates Surg..

[61] Lin Q (2015). Comparison of minimally invasive and open colorectal resections for patients undergoing simultaneous R0 resection for liver metastases: a propensity score analysis. Int J Colorectal Dis..

[62] Nachmany I (2015). Laparoscopic versus open liver resection for metastatic colorectal cancer. Eur J Surg Oncol..

[63] Tohme S (2015). Minimally Invasive Resection of Colorectal Cancer Liver Metastases Leads to an Earlier Initiation of Chemotherapy Compared to Open Surgery. J Gastrointest Surg..

[64] Lewin JW (2016). Long-term survival in laparoscopic vs open resection for colorectal liver metastases: inverse probability of treatment weighting using propensity scores. HPB (Oxford)..

[65] Ratti F (2016). Impact of totally laparoscopic combined management of colorectal cancer with synchronous hepatic metastases on severity of complications: a propensity-score-based analysis. Surg Endosc..

[66] Tranchart H (2016). Laparoscopic simultaneous resection of colorectal primary tumor and liver metastases: a propensity score matching analysis. Surg Endosc..

[67] Untereiner X (2016). Laparoscopic hepatectomy versus open hepatectomy for colorectal cancer liver metastases: comparative study with propensity score matching. Hepatobiliary Surg Nutr..

[68] Cipriani F (2016). Propensity score-based analysis of outcomes of laparoscopic versus open liver resection for colorectal metastases. Br J Surg..

[69] Guyatt GH (2008). GRADE: an emerging consensus on rating quality of evidence and strength of recommendations. BMJ..

[70] Gigot JF (2002). Laparoscopic liver resection for malignant liver tumors: Preliminary results of a multicenter European study. Ann Surg..

[71] Mala T, Edwin B (2005). Role and limitations of laparoscopic liver resection of colorectal metastases. Dig Dis..

[72] Kaneko H (2004). Hepatic resection using stapling devices. Am J Surg..

[73] Chang S, Laurent A, Tayar C, Karoui M, Cherqui D (2007). Laparoscopy as a routine approach for left lateral sectionectomy. Br J Surg..

[74] Simillis C (2007). Laparoscopic versus open hepatic resections for benign and malignant neoplasms—a meta-analysis. Surgery..

[75] Uchiyama K (2010). Combined use of contrast-enhanced intraoperative ultrasonography and a fluorescence navigation system for identifying hepatic metastases. World J Surg.

[76] Edna TH, Bjerkeset T (1998). Perioperative blood transfusions reduce long-term survival following surgery for colorectal cancer. Dis Colon Rectum..

[77] Mynster T, Christensen IJ, Moesgaard F, Nielsen HJ (2000). Effects of the combination of blood transfusion and postoperative infectious complications on prognosis after surgery for colorectal cancer. Danish RANX05 Colorectal Cancer Study Group. Br J Surg..

[78] Meng J (2013). Effects of allogeneic blood transfusion in patients with stage II colon cancer. Asian Pac J Cancer Prev..

[79] Li XX (2015). Effects of perioperative blood transfusion on the prognosis in hereditary and sporadic colon cancer. Biomarkers..

[80] D’Agostino RB (1998). Propensity score methods for bias reduction in the comparison of a treatment to a non-randomized control group. Stat Med..

[81] Lonjon G (2014). Comparison of treatment effect estimates from prospective nonrandomized studies with propensity score analysis and randomized controlled trials of surgical procedures. Ann Surg..

[82] Moher D, Liberati A, Tetzlaff J, Altman DG, Group P (2009). Preferred reporting items for systematic reviews and meta-analyses: the PRISMA statement. BMJ..

[83] Stang A (2010). Critical evaluation of the Newcastle-Ottawa scale for the assessment of the quality of nonrandomized studies in meta-analyses. Eur J Epidemiol.

[84] Xiong JJ (2013). Laparoscopic vs open total gastrectomy for gastric cancer: a meta-analysis. World J Gastroenterol..

[85] Parmar MK, Torri V, Stewart L (1998). Extracting summary statistics to perform meta-analyses of the published literature for survival endpoints. Stat Med..

[86] Hozo SP, Djulbegovic B, Hozo I (2005). Estimating the mean and variance from the median, range, and the size of a sample. BMC Med Res Methodol..

[87] Higgins JP, Thompson SG, Deeks JJ, Altman DG (2003). Measuring inconsistency in meta-analyses. BMJ..

[88] Xiong JJ (2014). Meta-analysis of pancreaticogastrostomy versus pancreaticojejunostomy after pancreaticoduodenectomy. Br J Surg..

[89] Sterne JA, Egger M, Smith GD (2001). Systematic reviews in health care: Investigating and dealing with publication and other biases in meta-analysis. BMJ..

