# Author Correction: Laparoscopic Versus Open Liver Resection for Colorectal Liver Metastases: A Comprehensive Systematic Review and Meta-analysis

**DOI:** 10.1038/s41598-018-24787-0

**Published:** 2018-04-18

**Authors:** Si-Ming Xie, Jun-Jie Xiong, Xue-Ting Liu, Hong-Yu Chen, Daniel Iglesia-García, Kiran Altaf, Shameena Bharucha, Wei Huang, Quentin M. Nunes, Peter Szatmary, Xu-Bao Liu

**Affiliations:** 1 Department of Gastrointestinal Surgery, West China Hospital of Sichuan University, Cheng du, China; 2 People’s Hospital of Deyang, Deyang, China; 3 Departments of Pancreatic Surgery, West China Hospital of Sichuan University, Chengdu, China; 4 Department of gastrointestinal Surgery, West China Hospital of Sichuan University, Chengdu, China; 5 Clinical Directorate of General Surgery, Royal Liverpool and Broadgreen University Hospitals NHS Trust, Liverpool, UK

Correction to: *Scientific Reports* 10.1038/s41598-017-00978-z, published online 21 April 2017

The original version of this Article contained an error in Affiliation 2 which was incorrectly listed as ‘People’s Hospital of Deyan, Chengdu and Deyang, Cheng du, China’. The correct affiliation is listed below:

People’s Hospital of Deyang, Deyang, China.

This error has now been corrected in the PDF and HTML versions of the Article.

