# Supplementary material for: Laparoscopic Versus Open Liver Resection for Colorectal Liver Metastases: A Comprehensive Systematic Review and Meta-analysis
Source: Sci Rep. 2017 Apr 21;7:1012. doi: 10.1038/s41598-017-00978-z (PMC5430829; doi:10.1038/s41598-017-00978-z)
Supplement: Supplementary file 1 — SUPPLEMENTARY INFO [file 41598_2017_978_MOESM1_ESM.pdf]

Laparoscopic Versus Open Liver Resection for Colorectal Liver Metastases: A Comprehensive Systematic Review and Meta-analysis

Si-Ming Xie[1,*], Jun-Jie Xiong[2,*], Xue-Ting Liu[1,*], Hong-Yu Chen[2], Daniel de la Iglesia-García[3], Kiran Altaf[4], Shameena Bharucha[4], Wei Huang[4], Quentin M. Nunes[4], Peter Szatmary[4] & Xu-Bao Liu[2]

Supplemental Fig.S1-Forest plots for secondary outcomes

A. Operation time

	LLR			OLR				Mean Difference	
Study or Subgroup	Mean	SD	Total	Mean	SD	Total	Weight	IV, Random, 95% CI	Year
Mala 2002	187	82.8	15	185	87	14	2.9%	2.00 [-59.91, 63.91]	2002
Castaing 2009	278	123	60	294	89	60	4.8%	-16.00 [-54.42, 22.42]	2009
Huh 2011	358	202.5	20	278	151.3	20	1.2%	80.00 [-30.78, 190.78]	2011
Chen 2011	350	45	23	342	38	18	6.1%	8.00 [-17.42, 33.42]	2011
Topal 2012	257.5	108.8	20	232.5	130	20	2.3%	25.00 [-49.29, 99.29]	2012
Hu 2012	313	44	13	350	46	13	5.2%	-37.00 [-71.60, -2.40]	2012
Inoue 2013	204	101	23	230	90	24	3.4%	-26.00 [-80.77, 28.77]	2013
Guerron 2013	239	17	40	219	16	40	7.7%	20.00 [12.77, 27.23]	2013
Cheung 2013	180	129.5	20	210	173.5	40	2.1%	-30.00 [-108.18, 48.18]	2013
Qiu 2014	230	60	24	280	75	25	4.8%	-50.00 [-87.95, -12.05]	2014
Montalti 2014	288	82	57	284	112	57	5.0%	4.00 [-32.04, 40.04]	2014
Jung 2014	290	183.5	24	244	131	24	1.7%	46.00 [-44.20, 136.20]	2014
Kubota 2014	333.9	150.3	43	305.9	107.1	62	3.6%	28.00 [-24.24, 80.24]	2014
Takasu 2014	472	90	7	466	107	7	1.4%	6.00 [-97.58, 109.58]	2014
Iwahashi 2014	377	29	21	369	31	21	6.9%	8.00 [-10.16, 26.16]	2014
Lin 2015	317.5	47.4	36	251.7	49.6	36	6.5%	65.80 [43.39, 88.21]	2015
Nachmany 2015	282	120	42	241	107	132	4.6%	41.00 [0.38, 81.62]	2015
Beppu 2015	282	166.7	171	277	230.5	342	5.1%	5.00 [-29.94, 39.94]	2015
de'Angelis 2015	210.5	80	52	180	90	52	5.4%	30.50 [-2.23, 63.23]	2015
Hasegawa 2015	228	110.2	100	277	157.5	68	4.3%	-49.00 [-92.22, -5.78]	2015
Cipriani 2016	295	137.5	133	210	247.5	133	3.9%	85.00 [36.88, 133.12]	2016
Untereiner 2016	230	120.5	18	180	100.5	18	2.4%	50.00 [-22.49, 122.49]	2016
Ratti 2016	420	148.3	25	310	115	50	2.7%	110.00 [43.70, 176.30]	2016
Tranchart 2016	332	110	89	308	86	89	5.8%	24.00 [-5.01, 53.01]	2016
**Total (95% CI)**			**1076**			**1365**	**100.0%**	**14.44 [1.01, 27.88]**	

Heterogeneity: Tau² = 596.59; Chi² = 78.44, df = 23 (P < 0.00001); I² = 71%

Test for overall effect: Z = 2.11 (P = 0.04)

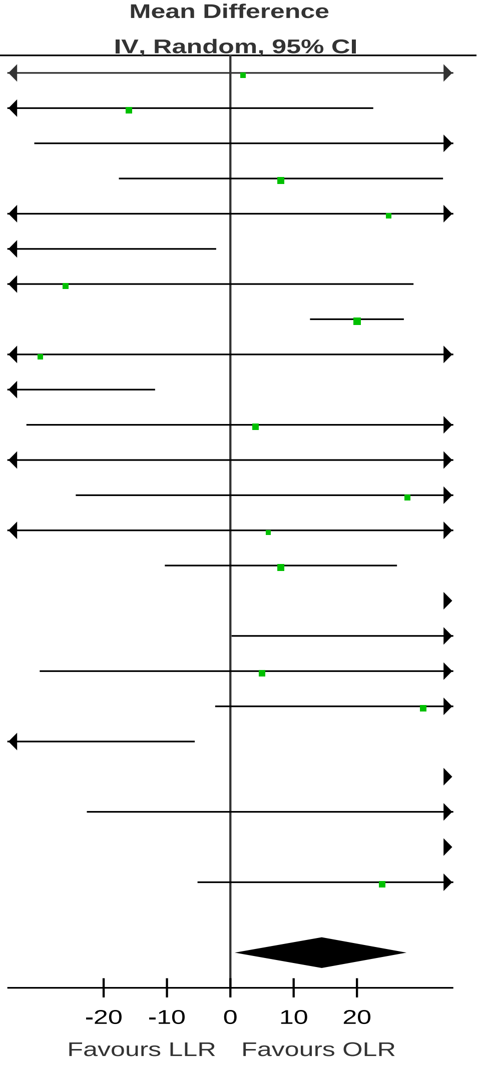

B. Intraoperative blood loss

Study or Subgroup	LLR Mean	LLR SD	LLR Total	OLR Mean	OLR SD	OLR Total	Weight	Mean Difference IV, Random, 95% CI	Year
Mala 2002	600	680	15	500	760	14	0.7%	100.00 [-426.22, 626.22]	2002
Chen 2011	275	96	23	590	85	18	5.0%	-315.00 [-370.51, -259.49]	2011
Huh 2011	350	267.5	20	500	325	20	3.0%	-150.00 [-334.48, 34.48]	2011
Topal 2012	550	1,025	20	550	650	20	0.7%	0.00 [-531.93, 531.93]	2012
Hu 2012	258	111	13	273	95	13	4.7%	-15.00 [-94.42, 64.42]	2012
Cannon 2012	202	180	35	392	324	138	4.6%	-190.00 [-270.49, -109.51]	2012
Guerron 2013	376	122	40	753	120	40	5.0%	-377.00 [-430.03, -323.97]	2013
Inoue 2013	99	207	23	397	381	24	3.1%	-298.00 [-472.33, -123.67]	2013
Cheung 2013	200	330	20	310	290	40	3.2%	-110.00 [-280.27, 60.27]	2013
Takasu 2014	152	128	7	496	191	7	3.2%	-344.00 [-514.33, -173.67]	2014
Montalti 2014	691.5	427	57	454.2	328	57	3.7%	237.30 [97.52, 377.08]	2014
Qiu 2014	210	170	24	380	165	25	4.4%	-170.00 [-263.86, -76.14]	2014
Iwahashi 2014	198	39	21	326	50	21	5.2%	-128.00 [-155.12, -100.88]	2014
Kubota 2014	287.3	459.3	43	579.3	392	62	3.2%	-292.00 [-460.42, -123.58]	2014
Jung 2014	325	237.5	24	250	225	24	3.8%	75.00 [-55.89, 205.89]	2014
Hasegawa 2015	127	487.2	100	620	568	68	3.3%	-493.00 [-658.36, -327.64]	2015
Nachmany 2015	251	305	42	422.7	410	132	4.1%	-171.70 [-287.46, -55.94]	2015
Tohme 2015	150	33.3	66	250	75	66	5.3%	-100.00 [-119.80, -80.20]	2015
Beppu 2015	163	559.2	171	405	1,873.3	342	2.6%	-242.00 [-457.50, -26.50]	2015
Lin 2015	278.1	173.3	36	382.5	145.6	36	4.7%	-104.40 [-178.34, -30.46]	2015
Langella 2015	100	143.7	37	85	151.2	37	4.8%	15.00 [-52.21, 82.21]	2015
de'Angelis 2015	200	100	52	300	225	52	4.8%	-100.00 [-166.92, -33.08]	2015
Ratti 2016	350	183.3	25	600	250	50	4.3%	-250.00 [-349.82, -150.18]	2016
Cipriani 2016	400	725	133	500	1,137.5	133	2.4%	-100.00 [-329.25, 129.25]	2016
Tranchart 2016	229	228	89	188	207	89	4.9%	41.00 [-22.98, 104.98]	2016
Untereiner 2016	30	41.8	18	200	86.8	18	5.1%	-170.00 [-214.51, -125.49]	2016
**Total (95% CI)**			**1154**			**1546**	**100.0%**	**-147.46 [-195.78, -99.15]**	

Heterogeneity: Tau² = 11415.51; Chi² = 271.11, df = 25 (P < 0.00001); I² = 91%

Test for overall effect: Z = 5.98 (P < 0.00001)

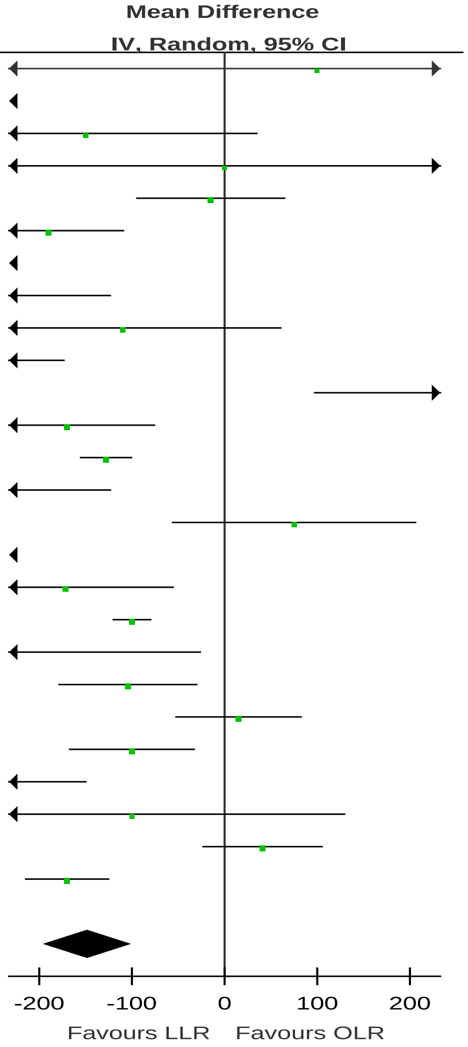

C. Blood transfusions

Study or Subgroup	LLR Events	LLR Total	OLR Events	OLR Total	Weight	Odds Ratio M-H, Fixed, 95% CI	Year
Castaing 2009	9	60	22	60	15.2%	0.30 [0.13, 0.74]	2009
Hu 2012	2	13	3	13	2.1%	0.61 [0.08, 4.41]	2012
Cannon 2012	6	35	44	138	12.0%	0.44 [0.17, 1.14]	2012
Guerron 2013	2	40	8	40	6.2%	0.21 [0.04, 1.06]	2013
Cheung 2013	0	20	1	40	0.8%	0.64 [0.03, 16.48]	2013
Inoue 2013	1	23	4	24	3.0%	0.23 [0.02, 2.21]	2013
Doughtie 2013	1	8	23	76	3.1%	0.33 [0.04, 2.83]	2013
Kubota 2014	1	43	13	62	8.4%	0.09 [0.01, 0.71]	2014
Langella 2015	0	37	2	37	2.0%	0.19 [0.01, 4.08]	2015
de'Angelis 2015	3	52	11	52	8.4%	0.23 [0.06, 0.87]	2015
Tohme 2015	4	66	3	66	2.3%	1.35 [0.29, 6.30]	2015
Beppu 2015	14	171	44	342	21.8%	0.60 [0.32, 1.14]	2015
Untereiner 2016	1	18	3	18	2.3%	0.29 [0.03, 3.14]	2016
Tranchart 2016	7	89	12	89	9.0%	0.55 [0.21, 1.46]	2016
Ratti 2016	2	25	7	50	3.5%	0.53 [0.10, 2.78]	2016
**Total (95% CI)**		**700**		**1107**	**100.0%**	**0.41 [0.30, 0.58]**	
Total events	53		200				

Heterogeneity: Chi² = 8.90, df = 14 (P = 0.84); I² = 0%

Test for overall effect: Z = 5.25 (P < 0.00001)

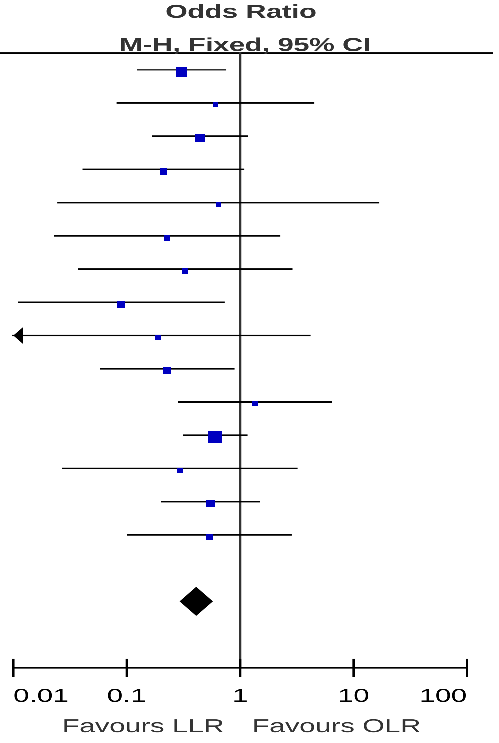

D. Overall morbidity

Study or Subgroup	LLR Events	LLR Total	OLR Events	OLR Total	Weight	Odds Ratio M-H, Fixed, 95% CI	Year
Mala 2002	2	13	4	14	0.8%	0.45 [0.07, 3.04]	2002
Castaing 2009	16	60	17	60	3.2%	0.92 [0.41, 2.05]	2009
Welsh 2010	56	266	227	886	21.0%	0.77 [0.56, 1.08]	2010
Nguyen 2011	1	24	3	25	0.7%	0.32 [0.03, 3.30]	2011
Chen 2011	8	23	9	18	1.7%	0.53 [0.15, 1.88]	2011
Huh 2011	10	20	8	20	1.0%	1.50 [0.43, 5.25]	2011
Cannon 2012	9	35	68	138	5.2%	0.36 [0.16, 0.82]	2012
Topal 2012	7	20	7	20	1.2%	1.00 [0.27, 3.67]	2012
Hu 2012	1	13	0	13	0.1%	3.24 [0.12, 87.13]	2012
Cheung 2013	2	20	2	40	0.3%	2.11 [0.27, 16.21]	2013
Guerron 2013	6	40	8	40	1.7%	0.71 [0.22, 2.26]	2013
Doughtie 2013	1	8	46	76	1.9%	0.09 [0.01, 0.80]	2013
Inoue 2013	2	23	5	24	1.1%	0.36 [0.06, 2.09]	2013
Takasu 2014	1	7	2	7	0.4%	0.42 [0.03, 6.06]	2014
Iwahashi 2014	2	21	5	21	1.2%	0.34 [0.06, 1.98]	2014
Montalti 2014	9	57	18	57	3.9%	0.41 [0.16, 1.00]	2014
Qiu 2014	6	24	12	25	2.2%	0.36 [0.11, 1.21]	2014
Jung 2014	4	24	10	24	2.1%	0.28 [0.07, 1.08]	2014
Kubota 2014	1	43	6	62	1.2%	0.22 [0.03, 1.92]	2014
Lin 2015	9	36	11	36	2.1%	0.76 [0.27, 2.13]	2015
Beppu 2015	24	171	43	342	6.3%	1.14 [0.66, 1.94]	2015
de'Angelis 2015	9	52	11	52	2.3%	0.78 [0.29, 2.08]	2015
Langella 2015	5	37	14	37	3.1%	0.26 [0.08, 0.81]	2015
Tohme 2015	17	66	25	66	4.7%	0.57 [0.27, 1.20]	2015
Nachmany 2015	11	42	54	132	4.9%	0.51 [0.24, 1.11]	2015
Cipriani 2016	31	133	53	133	10.3%	0.46 [0.27, 0.78]	2016
Untereiner 2016	5	18	7	18	1.3%	0.60 [0.15, 2.45]	2016
Ratti 2016	6	25	12	50	1.5%	1.00 [0.32, 3.08]	2016
Tranchart 2016	25	89	25	89	4.6%	1.00 [0.52, 1.92]	2016
Lewin 2016	25	140	35	122	7.8%	0.54 [0.30, 0.97]	2016
**Total (95% CI)**		**1550**		**2647**	**100.0%**	**0.64 [0.55, 0.75]**	
Total events	311		747				

Heterogeneity: Chi² = 29.02, df = 29 (P = 0.46); I² = 0%

Test for overall effect: Z = 5.48 (P < 0.00001)

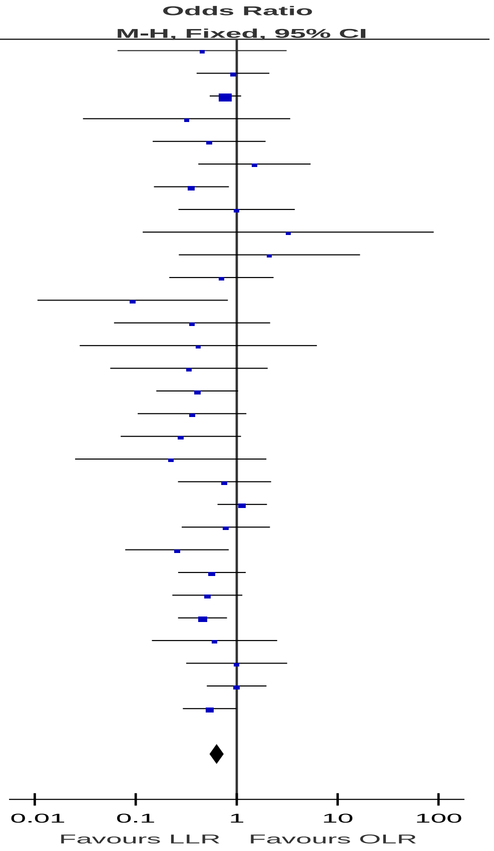

E. Mortality

	LLR		OLR			Odds Ratio	
Study or Subgroup	Events	Total	Events	Total	Weight	M-H, Fixed, 95% CI	Year
Mala 2002	0	13	0	14		Not estimable	2002
Castaing 2009	10	60	10	60	31.2%	1.00 [0.38, 2.61]	2009
Welsh 2010	3	266	12	886	20.5%	0.83 [0.23, 2.97]	2010
Huh 2011	0	20	0	20		Not estimable	2011
Topal 2012	0	20	0	20		Not estimable	2012
Hu 2012	0	13	0	13		Not estimable	2012
Cannon 2012	0	35	1	138	2.3%	1.29 [0.05, 32.37]	2012
Guerron 2013	0	40	0	40		Not estimable	2013
Doughtie 2013	0	8	4	76	3.3%	0.95 [0.05, 19.17]	2013
Inoue 2013	0	23	0	24		Not estimable	2013
Cheung 2013	0	20	0	40		Not estimable	2013
Kubota 2014	0	43	0	62		Not estimable	2014
Jung 2014	0	24	0	24		Not estimable	2014
Takasu 2014	0	7	0	7		Not estimable	2014
Montalti 2014	0	57	0	57		Not estimable	2014
Qiu 2014	0	24	0	25		Not estimable	2014
Beppu 2015	0	171	2	342	6.2%	0.40 [0.02, 8.32]	2015
Lin 2015	0	36	0	36		Not estimable	2015
Allard 2015	6	153	3	153	10.8%	2.04 [0.50, 8.31]	2015
Langella 2015	0	37	0	37		Not estimable	2015
Hasegawa 2015	1	100	1	68	4.4%	0.68 [0.04, 11.01]	2015
de'Angelis 2015	0	52	2	52	9.3%	0.19 [0.01, 4.11]	2015
Nachmany 2015	0	42	1	132	2.7%	1.03 [0.04, 25.79]	2015
Tohme 2015	0	66	0	66		Not estimable	2015
Cipriani 2016	1	133	2	133	7.4%	0.50 [0.04, 5.54]	2016
Untereiner 2016	0	18	0	18		Not estimable	2016
Ratti 2016	0	25	0	50		Not estimable	2016
Tranchart 2016	2	89	0	89	1.8%	5.11 [0.24, 108.06]	2016
**Total (95% CI)**		**1595**		**2682**	**100.0%**	**0.99 [0.58, 1.70]**	
Total events	23		38				

Heterogeneity: Chi² = 4.07, df = 10 (P = 0.94); I² = 0%

Test for overall effect: Z = 0.02 (P = 0.98)

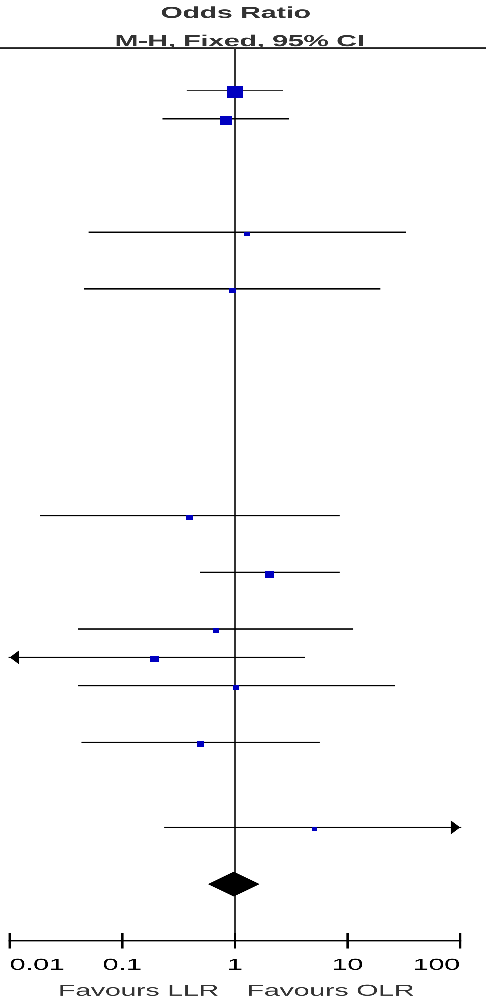

F. PLOS

	LLR			OLR				Mean Difference	
Study or Subgroup	Mean	SD	Total	Mean	SD	Total	Weight	IV, Random, 95% CI	Year
Mala 2002	4	1.8	13	8.5	7	14	2.4%	-4.50 [-8.30, -0.70]	2002
Castaing 2009	10	9.2	60	11	7.2	60	3.3%	-1.00 [-3.96, 1.96]	2009
Welsh 2010	10	6.5	266	10.5	8.4	866	7.2%	-0.50 [-1.46, 0.46]	2010
Chen 2011	12	1.5	23	16	2.5	18	6.4%	-4.00 [-5.31, -2.69]	2011
Huh 2011	10	9.3	20	10	9.5	20	1.2%	0.00 [-5.83, 5.83]	2011
Hu 2012	8.5	1.9	13	11.2	1.8	13	6.2%	-2.70 [-4.12, -1.28]	2012
Topal 2012	8	14	20	8	6	20	1.0%	0.00 [-6.68, 6.68]	2012
Cheung 2013	4.5	14.8	20	7	24.5	40	0.5%	-2.50 [-12.49, 7.49]	2013
Inoue 2013	10.8	11.2	23	13.9	10.3	24	1.1%	-3.10 [-9.26, 3.06]	2013
Guerron 2013	3.7	0.5	40	6.5	0.5	40	8.2%	-2.80 [-3.02, -2.58]	2013
Takasu 2014	16.2	6.1	7	36.1	24.9	7	0.1%	-19.90 [-38.89, -0.91]	2014
Qiu 2014	7.4	1.7	24	10.4	2.8	25	6.5%	-3.00 [-4.29, -1.71]	2014
Jung 2014	8	7	24	10.5	7.8	24	2.1%	-2.50 [-6.69, 1.69]	2014
Kubota 2014	7.3	1.8	43	14.2	7	62	5.3%	-6.90 [-8.72, -5.08]	2014
Montalti 2014	6.5	5	57	9.2	4	57	5.6%	-2.70 [-4.36, -1.04]	2014
Lin 2015	7.4	1.6	36	9	3.5	36	6.5%	-1.60 [-2.86, -0.34]	2015
Langella 2015	5	4	37	8	10	37	2.7%	-3.00 [-6.47, 0.47]	2015
Hasegawa 2015	9	43.3	100	16	25.8	68	0.4%	-7.00 [-17.47, 3.47]	2015
Nachmany 2015	8.4	5.6	42	6.7	2.7	132	5.4%	1.70 [-0.06, 3.46]	2015
Tohme 2015	4	1.5	66	5	1.7	66	7.9%	-1.00 [-1.55, -0.45]	2015
Beppu 2015	12	32.5	171	14	29.7	342	1.2%	-2.00 [-7.80, 3.80]	2015
de'Angelis 2015	6	2.5	52	9	4.7	52	6.1%	-3.00 [-4.45, -1.55]	2015
Untereiner 2016	6	3.3	18	7.5	4.5	18	3.9%	-1.50 [-4.08, 1.08]	2016
Cipriani 2016	4	14.5	133	7	38	133	0.9%	-3.00 [-9.91, 3.91]	2016
Ratti 2016	9	3.5	25	12	7	50	4.2%	-3.00 [-5.38, -0.62]	2016
Tranchart 2016	10.3	9.6	89	12.2	9.2	89	3.6%	-1.90 [-4.66, 0.86]	2016
**Total (95% CI)**			**1422**			**2313**	**100.0%**	**-2.36 [-3.06, -1.66]**	

Heterogeneity: Tau² = 1.54; Chi² = 113.62, df = 25 (P < 0.00001); I² = 78%

Test for overall effect: Z = 6.61 (P < 0.00001)

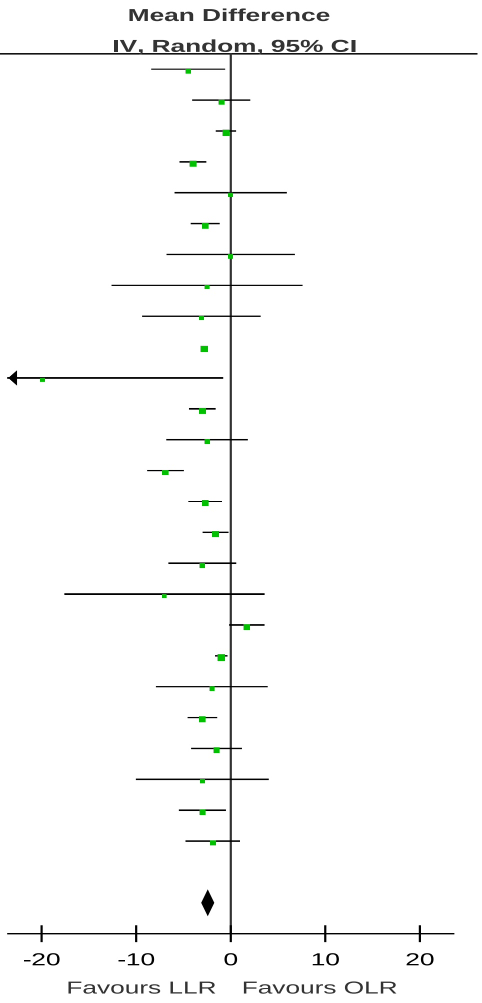

Supplemental Table S1 Quality assessment of included studies using modified Newcastle-Ottawa Scale

Authors	Selection[a]		Comparability[b]	Outcome assessment[c]		Quality score
	1	2	3	4	5	
Mala et al[27]	★	–	★★	★	★	★★★★★
Castaing et al[40e]	★	★	★★	★	★	★★★★★★
Welsh et al[41e]	★	–	★	★	★	★★★★
Chen et al[42]	★	★	–	★	★	★★★★
Huh et al[43e]	★	–	★★	★	★	★★★★★
Nguyen et al[20]	★	★	★	★	★	★★★★★
Cannon et al[44]	★	★	★★	★	★	★★★★★★
Hu et al[45]	★	★	★	★	★	★★★★★
Topal et al[46e]	★	★	★★	★	★	★★★★★★
Cheung et al[47e]	★	★	★	★	★	★★★★★

Doughtie et al[48e]	★	–	★★	★	★	★★★★★
Guerron et al[26e]	★	★	★	★	★	★★★★★
Inoue et al[49]	★	–	★★	★	★	★★★★★
Iwahashi et al[50e]	★	★	★★	★	★	★★★★★★
Jung et al[51e]	★	★	★	★	–	★★★★
Kubota et al[52]	★	★	–	★	★	★★★★
Montalti et al[53]	★	★	★★	★	★	★★★★★★
Qiu et al[54]	★	-	★★	★	★	★★★★★★
Takasu et al[55]	★	★	★	★	★	★★★★★
Allard et al[56e]	★	★	★	★	★	★★★★
Beppu et al[57]	★	★	★★	★	★	★★★★★★
de'Angelis et al [58e]	★	★	★★	★	★	★★★★★★
Hasegawa et al[59]	★	★	–	★	★	★★★★

Langella et al[60]	★	★	★	★	★	★★★★★
Lin et al[61e]	★	★	★★	★	★	★★★★★★
Nachmany et al[62]	★	★	–	★	★	★★★★
Tohme et al[63]	★	★	★	★	★	★★★★★
Lewin et al[64e]	★	★	★	★	★	★★★★★
Ratti et al[65e]	★	★	★	★	★	★★★★★
Tranchart et al[66e]	★	★	★	★	★	★★★★★
Untereiner et al[67e]	★	★	★★	★	★	★★★★★★
Cipriani et al[68e]	★	★	–	★	★	★★★★

[a] Selection: 1. Is the subject definition adequate or described? (if yes, one star); 2. Was the subject representative of the total population? (one star, if truly or obviously; no stars if subjects were selected group or not described).

[b] Comparability: Did the study have no differences between LLR and OLR for CCLM? Five main factors were considerate: positive node of primary tumor, disease-free interval, number of liver metastases, presence of liver tumor, CEA level. Other four factors: age, sex, ASA score, and pre- and postoperative chemotherapy were comparative (if yes, two stars; one star if there were no other differences between the two groups even if one or more of these five characteristics was not reported; no star was assigned if the two groups differed).

[c] Outcome assessment: Clearly defined outcome of interest (if yes, one star); Adequacy of follow-up (one star if less than 20% of CCLM patients lost to follow-up, otherwise no stars).

Supplemental Table S2 GRADE of evidence for the outcomes

**Outcomes**	**Illustrative comparative risks[a](95% CI)**		**Relative effect (95% CI)**	**No. of participants (studies)**	**Quality of the evidence (GRADE)**	**Comments**
	**Assumed risk**	**Corresponding risk**				
	OLR	LLR				
**Primary outcomes**						
Negative surgical margin	Study population		OR 1.61 (1.32 to 2.05)	4152 (24)	⊕⊕⊕⊝ moderate[2,13]	IMPORTANT
	865 per 1000 moderate	912 per 1000 (895 to 375)				
Recurrence	Study population 362 per 1000 328 per 1000 (284 to 375) moderate		OR 0.86 (0.70 to 1.06)	1850 (15)	⊕⊕⊕⊝ moderate[2,11]	CRITICAL
3-OS[b]	Moderate		HR 0.90 (0.78 to 1.04)	3899 (24)	⊕⊕⊕⊝ moderate[1,2,3,4,5]	CRITICAL
5-OS[c]	Study population 477 per 1000 456 per 1000 (412 to 500) moderate		HR 0.94 (0.82 to 1.07)	3452 (18)	⊕⊕⊕⊝ moderate[1,2,4,5,7]	CRITICAL
3-DFS[b]	Moderate		HR 0.89 (0.78 to 1)	2415 (19)	⊕⊕⊕⊝ moderate[1,2,4,5,6]	CRITICAL
5-DFS[c]	Study population 615 per 1000 604 per 1000 (556 to 657) moderate		HR 0.97 (0.85 to 1.12)	1399 (11)	⊕⊕⊕⊝ moderate[1,2,4,5,8]	CRITICAL
**Secondary outcomes**						
Operation time	The mean time in the intervention groups was 14.44higher(1.01 to 27.88 higher)		MD 14.44(1.01 to 27.88)	2441 (24)	⊕⊝⊝⊝ very low[2,8,9]	CRITICAL
Blood loss	The mean blood loss in the intervention groups was -147.46 lower(-195.78 to -99.15 lower)		MD -147.46 (-198.78 to -99.15)	2700 (26)	⊕⊝⊝⊝ very low[10]	IMPORTANT
Blood transfusion	Study population 181 per 1000	83 per 1000 (62 to	OR 0.41 (0.30 to 0.58)	1807 (15)	⊕⊕⊕⊝	IMPORTANT

	113) moderate				moderate[1,2]	
Overall morbidity	Study population 282 per 1000	201 per 1000 (178 to 228) moderate	OR 0.64 (0.55 to 0.75)	4197 (30)	⊕⊕⊕⊖ moderate[1,2]	IMPORTANT
Mortality	Study population 14 per 1000	14 per 1000 (8 to 24) moderate	OR 0.99 (0.58 to 1.70)	4277(28)	⊕⊕⊕⊖ moderate[1,2]	IMPORTANT
PLOS	The mean PLOS in the intervention groups were2.36 days lower (3.06 to 1.66 lower)		-	3735 (26)	⊕⊖⊖⊖ very low[12]	IMPORTANT

GRADE Working Group grades of evidence, *CI* confidence interval, *OLR* open liver resection, *LLR* laparoscopic liver resection, *OR* odds ratio, *OS* overall survival, *HR* hazard ratio, *DFS* disease-free survival, *PLOS* postoperative length of hospital stay.

[a]The basis for the assumed risk (e.g. the median control group risk across studies) is provided in footnotes;the corresponding risk (and its 95% CI) is based on the assumed risk in the comparison group and the relative effect of the intervention (and its 95% CI).

[b] Mean of follow-up: 3 years.

[c] Follow-up: 3-5 years.

⊕⊕⊕⊖ indicates moderate quality:further research is likely to have an important impact on our confidence in the estimate of effect and may change the estimate.

⊕⊖⊖⊖indicates very low quality:we are very uncertain about the estimate.

[1] The implementation process of outcomes are conducted stringently: quality level upgrading one level.

[2] Unmeasured confounding factors exist which reduce true effects and may lead to false effect: quality level upgrading one level.

[3] Heterogeneity test:p =0.78,I^2=0%, indicating no obvious inconsistency exists in 24 studies.

[4] The sample sizes and number of events more than the number of patients generated by a conventional sample size (n = 300) calculation for a single adequately powered trial, the overall imprecision for this outcome is not serious, thus not to downgrade the quality of evidence for imprecision.

[5] The funnel plot presents symmetric trend on 3-OS, and the risk of publication bias does not exist.

[6] Heterogeneity test: p = 0.99, I^2= 0%, indicating no obvious inconsistency exists in 18 studies.

[7] Heterogeneity test: p =0.51,I^2= 0%, indicating no obvious inconsistency exists in 11 studies.

[8]Heterogeneity test:p < 0.00001, I^2= 71%, indicating obvious inconsistency exists in 24 studies.

[9] Although the sample sizes and number of events (n = 2441) more than the number of patients generated by a conventional sample size (n = 300) calculation for a single adequately powered trial, the wide CI values (14.44 [1.01 to 27.88]) indicate uncertainty about the effect, thus not to upgrade the quality of evidence.

[10]Heterogeneity test:$p < 0.00001$, I^2=91%, indicating obvious inconsistency exists in 26 studies.
[11] Heterogeneity test: $p = 0.01$, $I^2 = 50\%$, indicating mild inconsistency maybe exist in 15 studies.
[12] Heterogeneity test: $p < 0.00001$, $I^2 = 78\%$, indicating serious inconsistency exists in 26 studies.
[13] Heterogeneity test: $p = 0.09$, $I^2 = 30\%$, indicating mild inconsistency maybe exist in 24 studies,but the overall inconsistency for this outcome is not serious, thus not to downgrade the quality of evidence.

Supplemental Table S3 Results of meta-regression analysis

		Coefficient (95%CI), *p*-value[a]					
		**Year of publication**	**Sex (men, %)**	**Age (year)**	**Study design**	**Tumor size**	**Adj R-squared[b](%)**
**Each covariate fitted into individual model**	**LLR versus OLR**						
	3-year OS	0.08 (0.01 to 0.14), 0.02	0.004 (-0.02 to 0.03), 0.67	-0.01 (-0.07 to 0.045), 0.68	0.22 (0.06 to 0.39), 0.01	-0.09 (-0.31 to 0.13), 0.39	
	5-year OS	0.01 (-0.05 to 0.07), 0.72	0.003 (-0.002 to 0.007), 0.32	-0.02 (-0.15 to 0.11), 0.0.70	0.06 (-0.09 to 0.22), 0.38	0.003 (-0.22 to 0.23), 0.97	
	Morbidity	-0.02 (-0.09 to 0.6), 0.59	0.001 (-0.02 to 0.02), 0.88	0.02 (-0.04 to 0.08), 0.45	0.10 (-0.15 to 0.36), 0.41	-0.05 (-0.26 to 0.15), 0.60	
**Each covariate fitted into multivariable model**	**LLR versus OLR**						
	3-year OS	0.05 (-0.13 to 0.23), 0.56	0.007 (-0.03 to 0.05), 0.69	-0.01 (-0.07 to 0.05), 0.75	0.02 (-0.62 to 0.66), 0.96	-0.06 (-0.42 to 0.31), 0.74	22.07
	5-year OS	0.02 (-0.13 to 0.19), 0.6754	-0.002 (-0.05 to 0.045), 0.92	-0.07 (-0.3 to 0.16), 0.48	-0.23 (-0.94 to 0.48), 0.45	-0.12 (-0.64 to 0.40), 0.59	8.16
	Morbidity	-0.08 (-0.19 to 0.04), 0.0.17	0.01 (-0.02 to 0.04), 0.40	0.03 (-0.03 to 0.09), 0.35	0.37 (-0.05 to 0.80), 0.08	0.18 (-0.12 to 0.49), 0.22	25.18

CI confidence interval, *LLR* laparoscopic liver resection, *OLR* open liver resection, *OS* overall survival.

[a]Two-sided *P* value.

[b]Proportion of variability among studies explained.
